# Microbial Communities of Polymetallic Deposits’ Acidic Ecosystems of Continental Climatic Zone With High Temperature Contrasts

**DOI:** 10.3389/fmicb.2019.01573

**Published:** 2019-07-17

**Authors:** Sergey N. Gavrilov, Aleksei A. Korzhenkov, Ilya V. Kublanov, Rafael Bargiela, Leonid V. Zamana, Alexandra A. Popova, Stepan V. Toshchakov, Peter N. Golyshin, Olga V. Golyshina

**Affiliations:** ^1^Laboratory of Metabolism of Extremophiles, Winogradsky Institute of Microbiology, FRC Biotechnology, RAS, Moscow, Russia; ^2^Laboratory of Bioinformatics, Genomics and Genome Editing, NRC Kurchatov Institute, Moscow, Russia; ^3^School of Natural Sciences, Bangor University, Bangor, United Kingdom; ^4^Laboratory of Geoecology and Hydrogeochemistry, Institute of Natural Resources, Ecology and Cryology, SB RAS, Chita, Russia; ^5^Centre for Environmental Biotechnology, Bangor University, Bangor, United Kingdom

**Keywords:** acid mine drainage (AMD) systems, mine-impacted environments, acidophilic bacteria, unclassified Euryarchaeota/Terrestrial Miscellaneous Euryarchaeotal Group (TMEG), *Ignavibacteriae*, Transbaikal area

## Abstract

Acid mine drainage (AMD) systems are globally widespread and are an important source of metal pollution in riverine and coastal systems. Microbial AMD communities have been extensively studied for their ability to thrive under extremely acidic conditions and for their immense contribution to the dissolution of metal ores. However, little is known on microbial inhabitants of AMD systems subjected to extremely contrasting continental seasonal temperature patterns as opposed to maritime climate zones, experiencing much weaker annual temperature variations. Here, we investigated three types of AMD sites in Eastern Transbaikalia (Russia). In this region, all surface water bodies undergo a deep and long (up to 6 months) freezing, with seasonal temperatures varying between −33 and +24°C, which starkly contrasts the common well-studied AMD environments. We sampled acidic pit lake (Sherlovaya Gora site) located in the area of a polymetallic deposit, acidic drainage water from Bugdaya gold-molybdenum-tungsten deposit and Ulan-Bulak natural acidic spring. These systems showed the abundance of bacteria-derived reads mostly affiliated with *Actinobacteria, Acidobacteria, Alpha-* and *Gammaproteobacteria*, chloroplasts, *Chloroflexi, Bacteroidetes*, and *Firmicutes*. Furthermore, candidate taxa “*Ca*. Saccharibacteria” (previously known as TM7), “*Ca*. Parcubacteria” (OD1) and WPS-2 were represented in substantial quantities (10–20%). Heterotrophy and iron redox cycling can be considered as central processes of carbon and energy flow for majority of detected bacterial taxa. Archaea were detected in low numbers, with Terrestrial Miscellaneous Euryarchaeal Group (TMEG), to be most abundant (3%) in acidic spring Ulan-Bulak. Composition of these communities was found to be typical in comparison to other AMD sites; however, certain groups (as *Ignavibacteriae*) could be specifically associated with this area. This study provides insight into the microbial diversity patterns in acidic ecosystems formed in areas of polymetallic deposits in extreme continental climate zone with contrasting temperature parameters.

## Introduction

Mining-impacted, pyrite-containing environments were extensively studied over past decades, and are known to host diverse Bacteria, Archaea, and Eukarya that drive elements transformations, especially those of carbon, iron, and sulfur (Johnson and Hallberg, 2008). Predominant bacterial species typically belong to *Proteobacteria*, in particular to Classes α and γ, and, more specifically, to the orders *Betaproteobacteriales* and *Acidithiobacillales* (early considered to be separate Classes) inside *Gammaproteobacteria*, according to Silva 132 taxonomy. Furthermore, some members of *Nitrospirae, Actinobacteria, Firmicutes*, and *Acidobacteria* were commonly detected in these environments along with *Bacteroidetes* and some representatives of uncultured candidate divisions (Mendez-Garcia et al., 2015). Archaeal diversity is predominantly represented by mesophilic members of families *Ferroplasmaceae, Cuniculiplasmataceae*, and some other archaea from the order *Thermoplasmatales* of the phylum *Euryarchaeota* without established taxonomic status (Golyshina, 2011; Golyshina et al., 2016, 2019; Korzhenkov et al., 2019). Further archaeal members in mine-impacted environments appearing in lower numbers, are represented by “*Candidatus* Micrarchaeota” and “*Ca*. Parvarchaeota”-related organisms (Baker et al., 2010; Golyshina et al., 2017), unclassified Euryarchaeota/Terrestrial Miscellaneous Euryarchaeal Group (TMEG), and some *Thaumarchaeota* (Falagán et al., 2014; Mendez-García et al., 2014).

Acidic pit lakes formed as an outcome of opencast mining and groundwater accumulation are widely distributed in mining areas. A significant body of research data has been produced on pit lakes located in the Iberian Pyrite Belt, where typical and atypical acid mine drainage (AMD) organisms were revealed (Falagán et al., 2014). Natural springs that actively discharge groundwater in acidic areas, and hence contribute to the hydro- and geochemistry in these settings may also be considered as AMD ecosystems, microbiology of which has earlier been reviewed (Johnson and Hallberg, 2003; Chen et al., 2016). Numerous natural and mining-impacted acidic sites of Eastern Transbaikalia, Russia, are located in the area of headwaters of the river Amur, more than 1,000 km eastwards of the lake Baikal, and represent a generous source of distinctive extreme environments. This area remains microbiologically under sampled with only a small number of recent studies available that were mostly dedicated to the diversity of sulfate-reducing bacteria (Karnachuk et al., 2015, 2016; Kadnikov et al., 2016 and others). The present work is focused on the elucidation of diversity of microbial inhabitants in three different types of acidic settings in this area. Sherlovaya Gora opencast pit lake located on the polymetallic deposit, acidic drainage water from pyrite/arsenopyrite Bugdaya deposit (Akatui group) and, finally, Ulan-Bulak natural acidic spring.

*Acidic Pit Lake at Sherlovaya Gora* (“Schorl Mountain” from Russian) is located in the area of a polymetallic deposit in south-eastern Transbaikalia and composed by cassiterite (SnO_2_), pyrites (FeS_2_), arsenopyrite (FeAsS), galena (PbS), sphalerite (ZnS), and chalcopyrite (CuFeS_2_). The mining in this area was mostly attributed to lead and beryl and was finished in 1995. Our study was particularly focused on opencast pit lake formed as a result of oxidative dissolution of sulfidic minerals (Banks et al., 2015) reflected in a relatively low pH (Table 1).

**Table 1:** Characteristics of samples used for barcoded 16S rRNA gene amplicon sequencing.

Sample ID	Description	Coordinates	pH	Temperature, °C	*E_h_*, mV
Shg4	Sherlovaya Gora Acidic Pit lake, upper layer/surface water	50°33′00.20″N, 116°16′07.8″E	3.23	17.3	+538
Shg1t	Sherlovaya Gora Acidic Pit lake/dense mats growing on surface of plants brunches immersed in water, 1 m depth	50°33′06.70″N, 116°16′05.34″E	2.83 (inside of mat) and 3.20 (on the surface of plant remnants)	18.0	+509
Shg13	Sherlovaya Gora Acidic Pit lake, bottom water, depth 29 m	50°33′00.20″N, 116°16′07.80″E	4.90	7.8	+220
Shg13sed	Sherlovaya Gora Acidic Pit lake/bottom sediment, depth 29 m	50°33′00.20″N, 116°16′07.80″E	4.90	7.8	+220
B3	Acidic drainage water in the area Bugdaya Mo-polymetallic deposit	51°09′29.85″N, 117°42′55.08″E	3.48	11.6	+511
UB1	Natural acidic spring Ulan-Bulak/small pond with red-brownish color	50°30′38.00′N, 118°55′04.90′E	2.37	15.0	+485
UB1sed	Natural acidic spring Ulan-Bulak/small pond with red-brownish color	50°30′38.00′N, 118°55′04.90′E	2.37	15.0	+485
UB2	Natural acidic spring Ulan-Bulak/small pond with reddish color	50°30′38.00′N, 118°55′04.90′E	2.36	16.8	n.d.

*Acidic drainage water from Bugdaya gold-copper* (*tungsten*) *polymetallic deposit* is located in the Eastern Transbaikalia area 310 km south-east from the Chita city on the north-western slope of a mountain ridge, dividing the basins of rivers Argun and Shilka, whose junction is the source of Amur river and at about 1,000 m altitude. The deposit is referred to Climax-type Porphyry Molybdenum deposits, but is characterized by a higher presence of gold. Main minerals are represented by pyrite, scheelite (CaWO_4_), molybdenite (MoS_2_), sphalerite, chalcopyrite, galena, fahlore [composed by tennantite (Cu_6_[Cu_4_(Fe,Zn)_2_]As_4_S_13_) and tetrahedrite (Cu_6_[Cu_4_(Fe,Zn)_2_]Sb_4_S_13_)] and natural gold. The main veined mineral is quartz, some mineral associations contain fluorite (Kovalenker et al., 2011).

*The Ulan-Bulak Urulyunguevsky natural spring* (hereinafter, Ulan-Bulak, “Red Spring” from Buryat) is a linearly represented source of surface waters on the bottom of the creek valley intensely colored reddish-orange with iron oxides. The total length of this system is about 600–700 m; it is situated on south-eastern slope of Nerchinsk mountain ridge. Previous geochemical analysis of waters revealed low pH with Ca and Mg being predominant cations (Zamana, 2013). Fe, Al, Mn, Ni, Zn, and Co were found as well. The chemical composition of waters was similar to that of acid mine drainage waters of Ural and Transbaikalia area. Sulfate was detected to be the predominant anion. Furthermore, sulfur compounds were represented by H_2_S (289.5 mg/L) and elemental S (0.83 mg/L). Weak spontaneous expulsions of CO_2_ were noted in warm seasons after melting of frozen layers of deposits (Zamana, 2013).

## Materials and Methods

### Sample Collection and DNA Extraction

Sampling of Sherlovaya Gora (Shg) Acidic Pit lake was done on 30.08.16. Samples selected for the assessment of microbial diversity were taken from: (1) upper layer/surface water, Shg4 (2) bottom layer water (30–50 cm above the sediment), Shg13, and (3) bottom sediment, Shg13sed. Bottom layers of the lake were sampled with a bathometer. Additionally, the sample of (4) dense mats growing on surface of herbaceous plants, immersed in lake water at a depth of about 1 m from the surface, Shg1t was taken as well.

Sampling of acidic drainage water seeping through brownish-black ores of **Bugdaya Au-Mo(W) polymetallic deposit (B3)** and forming a small pond was conducted on 05.09.16. The flow rate of seepage was calculated as ca. 20 ml/s. Water appeared as almost transparent with a low level of mineralization visualized. The sampling spot was also characterized by the presence of plant litter and dried out cyanobacterial mats.

**Figure 1:**
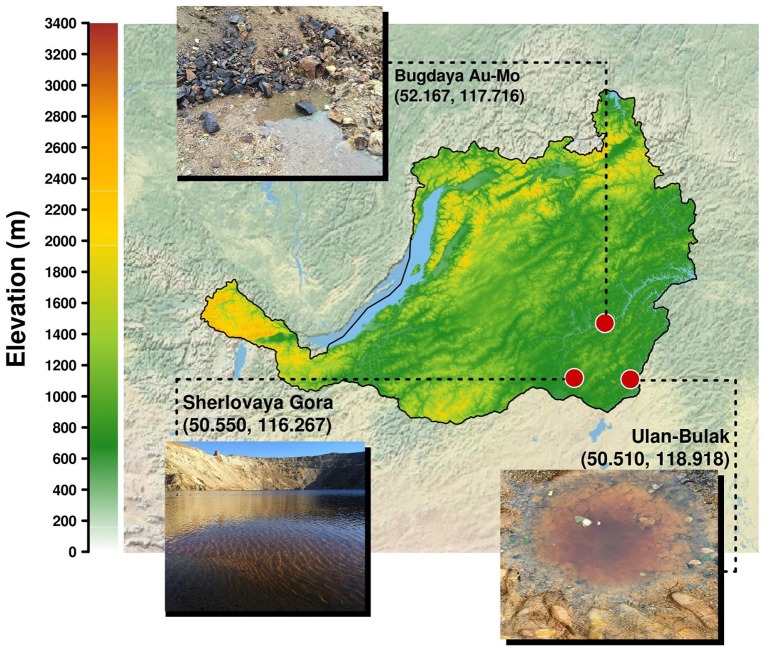
Localisation of sampling sites in Transbaikalia (the area to the east from the lake Baikal): *Acidic pit lake* (*site Sherlovaya Gora*), *Acidic drainage water from Bugdaya* Au-Mo (W) deposit and *Natural acidic spring Ulan-Bulak*.

On 03.09.16, samples were taken from two small ponds of the system **Natural acidic spring Ulan-Bulak (UB)** formed by active spring waters. UB1 is a small pond with red-brownish color having residues of different types of organic material, and UB2 is another small pond with significant amount of leaves and other plant residues on the bottom and intensively reddish colored water, characteristic for fulvic acids. The sediment with some organic material from UB1 (UB1sed) emplacement was also used for the study. The location of sampling sites is represented on the Figure 1. Main characteristics of these samples are summarized in the Table 1.

For DNA extraction from the surface water sample of Sherlovaya Gora pit lake and B3 sample from Bugdaya deposit, 1.2 L of water were filtered through Sterivex 0.2 μm pore size filter (Millipore). For the bottom water sample from Sherlovaya Gora and water samples from Ulan-Bulak, 0.2 L of water were filtered (sample volume in these cases was restricted by water turbidity which caused filter clogging). For each sedimental sample, 0.75 ml of wet sediment (or microbial foulings) were sampled, which roughly corresponds to 1.2 g in weight. The samples were routinely fixed with 50% sterile ethanol on-site for further transportation to the laboratory and DNA isolation. DNA was extracted from sediments using PowerSoil^®^ Isolation Kit (MO BIO Laboratories) according the manufacturer’s instructions. DNA extraction from Sterivex filters was performed using Meta-G-Nome™ DNA Isolation Kit (Epicenter) according the manufacturer’s instructions. All extraction experiments were performed in duplicates.

### Chemical Analysis

On-site measurements were performed for oxygen content – with Multi 340i handheld multimeter (WTW, Germany), рН – with portable precision pH meter CG 837 (Schott Instruments, Germany), *E_h_* (redox potential) and temperature – with HI 98120 ORP and temperature meter, electric conductivity – with HI 9033 (Hanna Instruments). For the sediment of Sherlovaya Gora lake, pH, *E_h_* and temperature were measured in a subsample inside bathometer immediately after buoying it up. The measurements were performed by immersing an appropriate sensor (pH, ORP or temperature) into the sediment on a depth of >1 cm, which provided full coverage of ceramic reference junction and glass membrane or platinum sensor of pH or ORP electrodes with wet sediment. For chemical analyzes, all water samples were filtered through ashless paper filters with 2–3 μm pore size. For cations, the samples were acidified with chemically pure nitric acid to pH 2.0. In the filtered samples, Ca, Mg, Al, Fe, Mn, Co, Ni, Cu, Zn, Sr, Cd, and Pb were quantified with flame atomic absorption spectroscopy (FAAS), Na and K – with flame emission spectroscopy, on a SOLAAR M6 spectrometer (Thermo Scientific). Sulfate ions were quantified by titration with BaCl_2_ in the presence of nitrochromazo dye as the indicator; fluoride – by potentiometry with corresponding ion-selective electrode in acetate-citrate buffer; chloride – by mercurimetry; Si, total phosphorus and nitrogen species (NO_3_^−^, NO_2_^−^, and NH_4_^+^) – by spectrophotometry. For ICP MS analyzes of elemental composition, water samples were sequentially filtered through ashless paper filters and 0.45 μm membrane filters, and final filtrates were commercially analyzed in Vinogradov Institute of Geochemistry SB RAS (Irkutsk, Russia) on an Element-2 mass spectrometer (Finnigan MAT) using the NSAM method No. 480X according the manufacturer’s instructions.

### 16S rRNA V3-V4 Amplicon Metabarcoding

Libraries of 16S rRNA amplicons corresponding to V3–V4 variable region were prepared by single PCR with double-indexed fusion primers as described by Fadrosh et al. (2014). 16S rRNA annealing of forward Pro341F (modified) and reverse 806R primers corresponded to 5′CCTAYGGGBGCWSCAG and 5′GGACTACHVGGGTWTCTAAT (Merkel et al., 2015).

Single-step PCR amplification was performed by qPCRmix-HS™ SYBR mastermix (Evrogen, Russia) using the following conditions: 30 cycles of denaturation at 95°C for 15 s; primer annealing at 58°C, 15 s; DNA elongation at 72°C, 25 s, followed by final DNA elongation for 5 min at 72°C. Purification of PCR products was done using the Cleanup Mini kit (Evrogen, Russia). The quality of the final libraries was assessed using the electrophoresis in agarose gel.

Libraries were sequenced with MiSeq™ Personal Sequencing System technology of Illumina Inc. (San Diego, CA, USA) using paired-end 250-bp reads. Demultiplexing was performed as described previously (Fadrosh et al., 2014). After demultiplexing, all reads were subjected to stringent quality filtering, and parts of reads, corresponding to 16S rRNA primers were removed using CLC Genomics Workbench 10.0 (Qiagen, Germany). OTU picking and taxonomy assignment was made using QIIME in open-reference mode (Caporaso et al., 2010) and Silva132 database (Quast et al., 2012), all parameters were set to default values.

### Data Availability

The 100 most abundant OTUs obtained from the analysis of high-throughput 16S rRNA profiling experiments were submitted to GenBank under accession numbers MK521704 – MK521803. Sequencing reads were submitted to MG-RAST server (Keegan et al., 2016) as a project #mgp88315 and are available by the following link: https://www.mg-rast.org/linkin.cgi?project=mgp88315.

## Results and Discussion

### Sherlovaya Gora

We have sampled the opencast pit lake of Sherlovaya Gora deposit. According to long-term monitoring of the Institute of Natural Resources, SB RAS, the lake remains presumably holomictic during the last decade with occasional mixing periods in spring or autumn seasons, it is 29 m-deep and is filled by ground- and surface waters. The level of water in the lake was recorded to be almost constant. The lake remains frozen during the winter period for about 6 months, and the ice depth is usually between 1.2 and 1.4 m with a temperature under the ice level being −0.4°C and about +4.1°C at the depth of 14 m. The area around the lake is covered by iron and manganese oxides and deposits of sulfates. Our chemical analysis showed that the concentration of sulfate ions was overwhelmingly high in this system (4,750 mg/L). Cations of the following metals were detected at total concentrations (mg/L): Ca (694), Zn (658), Fe (347), Mg (274), Mn (110), and Al (22.2). Additionally, many minor and trace elements were detected. Interestingly, F was detected at 15.1–23.4 mg/L, which is likely to be attributed to the greisen mineralization. *E_h_* distribution showed the gradient from +567 mV in the upper layer of the lake to +353 in the middle, and +266 on the bottom. Oxygen was found to be present in upper part of the lake only with a measured concentration 0.44 mg/L. Maximum content of ammonium NH_4_^+^ (3.59 mg/L) was found in the middle layer of water at ca. 15 m depth (Supplementary Table S1).

The 16S rRNA gene amplicon sequencing data showed minor archaeal (less than 0.5%), but mainly bacterial signatures in all studied sampling points of Opencast Pit Lake on the deposit Sherlovaya Gora.

Surface water amplicon sequencing dataset was predominantly derived from chloroplasts from several phototrophic eukaryotes, mainly, from mixotrophic freshwater microalgae of *Epipyxis* genus (39.4% of reads). Small flocks, characteristic to these golden algae, were visible in the surface water. Bacterial sequences were affiliated with “*Ferrovum*” (24.6%) and *Acidiphilium* genera (22.9%) and small proportions to uncultured organisms within the family *Acetobacteraceae* of *Alphaproteobacteria* (4.7%). “*Ferrovum*,” shown to be widespread in extremely acidic ferruginous mine waters and containing the only cultured species “*Ferrovum myxofaciens*,” is an extremely acidophilic, psychrotolerant obligate chemolithoautotroph and diazotroph, which utilizes ferrous iron as the only electron donor (Johnson et al., 2014). Heterotrophy together with certain photosynthetic (Kishimoto et al., 1995) and iron(III)-reducing activity with the expenditure of a variety of organic substrates indicate ecological significance of another genus, *Acidiphilium*, important in the biogeochemical cycling of iron at oxic-anoxic interfaces in acidic environments. A number of potentially hydrolytic enzymes were reported previously for *Acidiphilium* spp., pointing at their role in carbon (organic polymers) turnover (Ullrich et al., 2015). All other reads from surface water of Sherlovaya Gora pit lake pointed at various taxonomic groups represented in minor quantities (<1%). Among them worth noting are the sequences affiliated with *Acidicapsa* genus, whose members are capable of Fe(III) reduction and wood polysaccharides degradation, (Kulichevskaya et al., 2012; Falagán et al., 2017). Facultative anaerobic heterotrophic or/and facultative phototrophic iron-reducing species of the genus *Acidiphilium* and other *Acetobacteraceae* were previously detected as the most abundant phylotypes in pit lakes of the Iberian Pyrite Belt (Falagán et al., 2014; González-Toril et al., 2014), accounting for almost 80% of total reads in the upper layers (Santofimia et al., 2013).

Microbial mats developed on allochthonous organics (plant material remnants), are grown at similar temperature, pH and *E_h_* values as in the surface water (Supplementary Table S1). Bacteria-derived SSU rRNA gene reads predominantly (23.7%) corresponded to various uncultured *Acidimicrobiia*, of them, 14.2% were related to IMCC26256 deep branch comprised of bacteria isolated from artificial freshwater lake Soyang (South Korea). Other 1.3% of acidimicrobial reads were related to genus *Acidithrix* and the remaining 8.2% to four distinct uncultured actinobacteria unassigned at the family level. Members of the family *Acidimicrobiaceae* within the order *Acidimicrobiales* are recognized heterotrophic iron-metabolizing acidophiles, previously identified in pit lakes (Santofimia et al., 2013). Further major taxa, represented in the microbial mats were *Ktedonobacteraceae*-related chloroflexi (16.5%), which was a minor component in the surface water community (<0.1%), and chloroplasts from various organisms, which totally account for 16.0% but are distinct from the chloroplasts clade predominant in the surface water. Interestingly, *Ktedonobacteria* are soil-borne mesophilic and thermophilic heterotrophic hydrolytic filamentous microorganisms with one of their representatives, *Ktedonobacter racemifer*, possessing a 13 Mbp genome, currently known as one of the largest among prokaryotes (Chang et al., 2011). Recently, Yabe et al. (2017a) documented a wide distribution of *Ktedonobacteraceae* related clones in terrestrial environments, including moderately acidic ones. These same authors also described a representative of *Ktedonobacteraceae* family able to hydrolyse xylan and cellulose, major components of plant biomass (Yabe et al., 2017b). Considering this physiological feature, persistence of *Ktedonobacteraceae*-related phylotype in surface water and high abundance of this phylotype in the immersed plant-associated microbial mat, we could speculate that ktedonobacteria might be active organic matter decomposers in the pit lake ecosystem. Among other major groups detected in mats were *Acidobacteriaceae* Subgroup 1-related sequences comprising totally 12.1% of reads, including 2.3% from polysaccharidolytic *Acidicapsa* representatives and 9.8% related to various isolates unassigned at the genus or family level. Notably, the abundance of *Acidicapsa* increased threefold in the mats comparing to the surface water. Also, 6.3% reads from mats were affiliated with *Granulicella paludicola*, aerobic acidophilic heterotrophic bacteria hydrolysing polysaccharides (Pankratov and Dedysh, 2010). In the surface water, *Granulicella*-related sequences were rare (<0.02%). Uncultured *Acetobacteraceae* (5.2%) showed similar abundance as in surface water, while representatives of genus *Acidiphilium*, were twentyfold less represented compared to the surface water (1.2 vs. 22.9%). Also, 3.2% of bacterial WPS-2 clade-related signatures, identified earlier in contaminated soils and acidic environments (García-Moyano et al., 2012) and *Beijerinckiaceae*-related alphaproteobacteria (3.3%) were found in the mats formed on plant remnants. Worth mentioning are the sequences related to *Leptospirillum* genus (2.2%) of iron-oxidizing *Nitrospirae* and 1.6% sequences of CPla-3 termite group belonging to *Phycisphaerae* (*Planctomycetes*). Facultative anaerobes of *Phycisphaerae* class were isolated from freshwater and hypersaline lakes (Fukunaga et al., 2009; Kovaleva et al., 2015; Edwardson and Hollibaugh, 2018). The phylotypes related to *Beijerinckiaceae, Leptospirillum*, and *Phycisphaerae* were also detected in surface water, but comprised a negligible part of its microbial diversity (each less than 0.1%).

The deeper samples of Sherlovaya Gora pit lake were characterized by higher pH (4.9) and lower *E_h_* (+220 mV) values in comparison to the surface layers (Supplementary Table S1). Microbial composition of the community from the bottom of the lake showed a certain similarity with those from upper water samples and microbial mats. However, a significant downshift in abundance of reads derived from phototrophs, which could only be detected at 0.5% as uncultured *Chloroflexi*, was observed. Instead, the microbial diversity in bottom water showed predominance of obligate chemolithoautotrophic iron oxidizers of “*Ferrovum*” genus (12%), and representatives of uncultured *Acetobacteraceae* (15.5%), *Acidimicrobiia* (6.3%), *Ignavibacteriales* (9.7%), and *Elusimicrobia* (5.2%), potentially harboring organotrophic microorganisms. *Ignavibacteriales* were represented by one major and two minor phylotypes of uncultured organisms. Surprisingly, such a high abundance of ignavibacteria was detected in a cold (7.6°C) acidic environment far eastwards of the Rift Zone 2,000 km long plate boundary centered beneath Lake Baikal. However, the majority of *Ignavibacteriae*-related environmental clones, detected previously, came from subterrestrial sources or deep-sea sediments, and the only two cultured species of this phylum were isolated from thermal habitats (Iino et al., 2010; Podosokorskaya et al., 2013). The two known cultured ignavibacteria are facultatively anaerobic organotrophs that use various carbon sources. One of them, *Melioribacter roseus*, possesses а high hydrolytic potential along with capabilities of arsenate, nitrite and Fe(III) reduction (Podosokorskaya et al., 2013; Gavrilov et al., 2017), suggesting a possible involvement of *Ignavibacteriae*-related organisms in iron, nitrogen and mixed valence elements cycling in Sherlovaya Gora lake. Clones belonging to the class and phylum of the same name, *Elusimicrobia*, have been previously detected in various environments with high organic matter content, as well as in the groundwater of a deep subsurface gold mine (Magnabosco et al., 2016). The only cultured representative of this taxon is a strictly anaerobic monosaccharide-fermenting ultramicrobacterium isolated from gut of scarab beetle (Geissinger et al., 2009). Among less abundant phylotypes in the bottom water of the lake(1–5%), were betaproteobacteria of the genus *Sulfuricella* (1.1%); WPS-2 clade-related clones (4.4% of total reads), which have also been recovered from microbial mats; *Sporichthyaceae* family (2.6%, two phylotypes); clones related to Fe(III)-reducing acidobacteria of *Acidicapsa* genus (2.3%), *Rhodanobacter* genus of order *Xanthomonadales* (2.4%), as well as representatives of uncultured “*Ca*. Saccharibacteria” (candidate division TM7, 0.9%). The genus *Sulfuricella* contains the only species of sulfur-oxidizing, nitrate reducing facultative anaerobes (Kojima and Fukui, 2011). Representatives of *Sporichthyaceae* family are widespread in soils and aquifers, including cold environments, grow heterotrophilcally using soil humic acids as the only carbon source (Tamura, 2014). *Rhodanobacter* species are heterotrophic, facultatively anaerobic denitrifiers populating areas with neutral to moderately acidic pH, such as soil, terrestrial subsurface sediments or mine wastewaters, with some of species being able to degrade polysaccharides and oxidize thiosulfate (Green et al., 2012; Koh et al., 2015).

Among minor (less than 1%) community components of the bottom water of Sherlovaya Gora pit lake representatives of sulfur and As(III) oxidizing betaproteobacteria (*Thiomonas* genus), iron oxidisers of the genera *Acidithiobacillus* and *Acidithrix* (whose maximal abundance was detected in microbial mats on plant remnants), and heterotrophic iron-reducers of the genus *Metallibacterium* are worth noting. Facultatively anaerobic metallibacteria were found earlier to be the one of predominant groups of organisms in the sediments of acidic pit lake Cueva de la Mora, Iberian Pyrite belt, Spain (Falagán et al., 2014).

**Figure 2:**
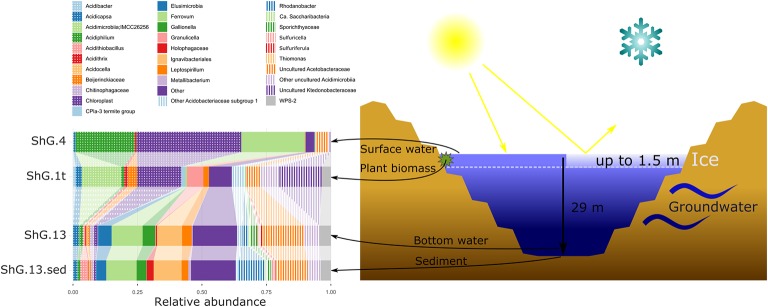
Microbial community composition of Sherlovaya Gora pit lake at different microniches according to 16S rRNA gene profiling. Shg4, surface water; shg1t, microbial mat on plant residues at ca. 1 m depth; shg13, bottom water at ca. 29 m depth; shg13.sed, bottom sediment. “Other” group includes: Archaea, other *Acidobacteria*, other *Actinobacteria*, other *Chloroflexi*, “*Candidatus* Dependentiae” (TM6), *Firmicutes, Gemmatimonadetes, Planctomycetes*, other *Proteobacteria*, Candidate Phyla Radiation (CPR) bacteria, *Verruccomicrobia* without distinct prevalence of any of them.

**Figure 3:**
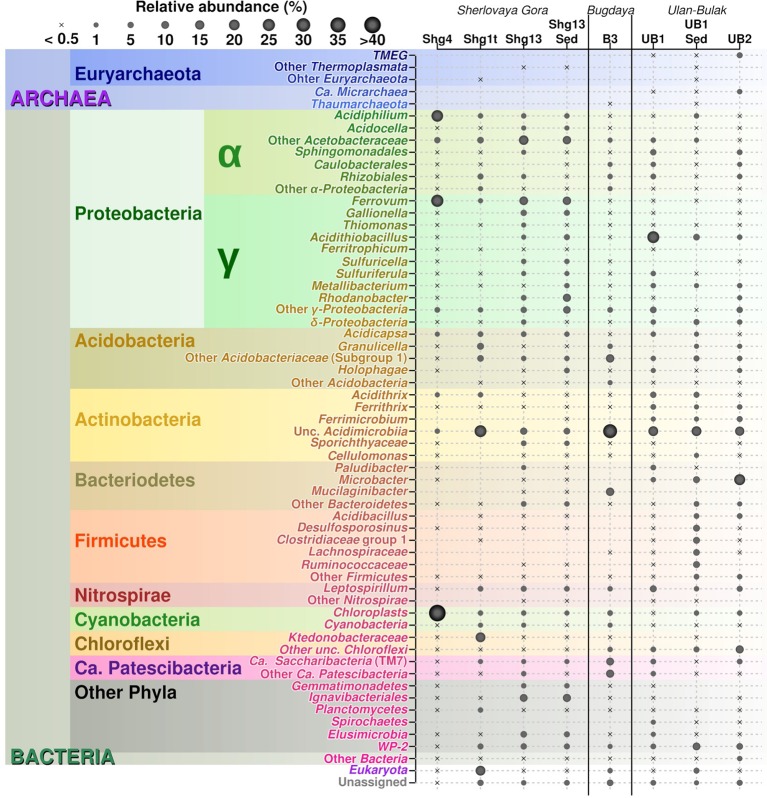
Relative abundances of 16S rRNA gene V3-V4 amplicon reads in studied sites.

Major taxa of pit lake sediments were similar, to those in bottom water (Figure 2) except for slightly decreased numbers of sequences related to uncultured *Acetobacteraceae* (11.8%), and various iron cycling organisms, making up to 9.8% altogether. The latter group was mainly represented by iron-oxidizing acidithiobacilli (3.1%), *Gallionella* spp. (3.8%) and *Leptospirillum* spp. (2.6%), as well as by minor numbers of *Ferrithrix* and *Acidithrix* (Figure 3). Organisms related to family *Acetobacteraceae*, including various heterotrophs, were the most abundant in the sediment and mainly represented by uncultured taxa with minor proportions of *Acidiphilium* and *Acidocella*. The abundance of *Ignavibacteriales*-related clones has been very similar, both in the sediment and bottom water (10.7 and 11.2%). The sediments’ third most abundant group (11.1% reads) was attributed to *Xanthomonadales* with 10.1% of *Rhodanobacter*-related and 1.0% of *Metallibacterium*-related signatures. Representation of *Elusimicrobia* (3.8%), *Acidicapsa* (1.6%), *Acidimicrobiia* (totally 4.9%) and WPS-2 clade (totally 3.8%) decreased in the sediment as compared to bottom water, whereas *Holophagaceae* representation increased to 2.5%. Within the minor part of the sediment community, of interest are the members of the family *Chitinophagaceae* (1.3%) with high hydrolytic potential. Several taxa, containing sulfur oxidizers of genera *Thiobacillus, Sulfuricella, Sulfuriferula* (all, betaproteobacteria) were also present in the sediments, each in minor quantities, but their total share was considerable (1.9%). To sum up, the surface water, which is warmer, more oxygenated and illuminated, was less diverse in comparison to bottom sediment and bottom water samples (Figure 2).

Ultimately, metabolic traits in microbial inhabitants of Sherlovaya Gora pit lake might be considered similar to other organisms from acidic pit lakes, where biogeochemical cycles of carbon and iron are linked together *via* the activity of phototrophic Fe(II) oxidizers and chemoheterotrophic Fe(III) reducers, mineralizing allochtonous, as well as autochtonous organics. Still, considerable proportion of allochtonous organics is likely to be decomposed by oxygen respiring or fermentative organisms in aerobic and anaerobic zones of the lake, respectively, while oxygenic phototrophs could significantly contribute to autochtonous organics production. Our chemical data suggest rather high soluble iron availability in the system (347 mg/L), part of which may also be adsorbed but easily available on the surface of iron minerals from underlying bedrock of Sherlovaya Gora pit lake. Dopson et al. (2008) observed inhibition of microbial Fe oxidation due to fluoride toxicity (F content detected to be in 15.1–23.4 mg/L in Sherlovaya Gora). Falagán et al. (2014) also noted a poor availability of phosphorus in acidic, highly stratified, iron-containing water systems, where ferric phosphate is extremely insoluble and phosphate availability was directly linked with redox variations of iron. There, the phosphate was found to be at higher concentrations in the anaerobic monimolimnion zone in comparison to the aerobic mixolimnion. One could also suggest that a part of microbial community might be associated with clay mineral formation, as Sánchez-España et al. (2018) earlier showed in acidic pit lake ecosystem in the Iberyan Pyritic Belt. However, relatively high abundance of iron cycling organisms in Sherlovaya Gora pit lake indicates the possibility of active Fe(II) and Fe(III) compounds turnover in the system.

Despite the high availability of sulfate ions in Shg samples, currently known bacteria responsible for sulfur compounds transformation appeared to be poorly represented in the site. We have only detected sequences of acidophilic sulfate reducing bacteria (SRB) of *Desulfosporosinus* genus, in minor numbers (0.3%, Figure 3) – from anoxic sediments of Sherlovaya Gora acidic pit lake. Similar situation was observed in Cueva de la Mora and explained by a probable loss in competition by SRB to heterotrophs for electron donors (small molecular weight organic molecules) (Falagán et al., 2014). On the other hand, a large pool of uncultured organisms and newly recognized groups without any background physiological information may also be potentially engaged in sulfur compounds transformations as well as other functions in Sherlovaya Gora pit lake.

Many similarities between acidic pit lakes of Sherlovaya Gora and Cueva de la Mora became apparent. However, while the seasonal fluctuations in mixolimnion of the Iberian Pyritic Belt were described as rather insignificant (10–25°C) (Falagán et al., 2014), huge differences in seasonal temperature regimes are characteristic for continental climate zone of Transbaikalia. Acidic pit lake remains frozen from mid-November till mid-May, which is the case for all water bodies in this area. Water temperature decreases down to −0.4°C under the ice sheet, which is more than a meter thick. These conditions increase oxygenation of the upper layer of the lake but hinder gas circulation and decrease availability of sun light as the energy source, thus significantly changing the community composition for almost half a year. In this view, the high abundance of phototrophs in surface water of the lake and presence of typical aerobic organisms in the bottom water in summer point at a good fitness and resilience of the microbial community to resist sharp changes in physico-chemical conditions of the environment. In relation to that, the activity of acidophiles at low temperatures was reported for Arctic, Antarctic and northern latitudes’ areas but no detailed information on characteristics of bacterial populations was provided (Dold et al., 2013; Wu et al., 2013).

According to the present study, moderate acidophiles outnumber extremely acidophilic counterparts in Sherlovaya Gora acidic pit lake. This suggests a discrepancy with previous data of Kadnikov et al. (2016), who studied the composition of microbial communities from another emplacement within Sherlovaya Gora ecosystem, in the wells drilled in the body of ore deposits. We consider that the higher abundance of iron and lower pH values in wells together with their depth and wellheads dimensions (Kadnikov et al., 2016) restrict mass and heat exchange with the surrounding environment. That may smoothen sharp seasonal variations in physico-chemical parameters of the ecosystem and thus shift the abundances of principal microbial taxa in the wells toward more extremely acidophilic iron-transforming organisms.

### Acidic Drainage Water Outlet From Bugdaya Au-Mo(W) Polymetallic Deposit

Main physico-chemical characteristics of water from this sample are summarized in Table 1 and Supplementary Table S2. The sampling site was also characterized by the presence of old leafs and dry crusts of cyanobacterial mats contributing to the organic pool in this ecosystem.

The chemical content of water was found to be typical for acid mine drainage waters of Transbaikalia, with high concentrations of sulfates and metals, particularly Al (Zamana, 2011; Zamana and Chechel, 2014). From other anions, high concentration of F^−^ (5.0% of all anions, Supplementary Table S2) was detected, which is originated from formation of complex compounds with Al (Zamana and Bukaty, 2004) and is characteristic for AMD of Transbaikalia, especially if fluorite is presented in ores. Main metal cation was found to be Ca (32.6% of all metals detected), followed by Mn (26.3%), and Al (17.9%, Supplementary Table S2). Low concentration of iron might be explained by precipitation of it as crust-like oxides of Fe, less of Mn, Al and Si on stones surfaces, so-named “sunburn of desert”. Fe was found to be represented rather in ferrous state (Fe^2+^). CO_2_ was detected either (Supplementary Table S2).

Drainage water percolating deposits was found to contain mostly bacterial sequences, however a minor fraction of reads (0.4%) attributed to *Thaumarchaeota* “*Candidatus* Nitrosotaleaceae” family were also detected. The largest numbers of bacterial reads in B3 sample (28.8%) were affiliated with *Acidimicrobiia* (mostly with uncultured organisms including 9.6% reads related to IMCC26256, abundant in Shg1t sample, but with a small proportion (0.4%) of signatures related to *Ferrithrix* genus of iron oxidizers and reducers, Figure 3). The second most abundant group in Bugdaya sample was represented by uncultured phylum-level bacterial lineage “*Ca*. Patescibacteria,*”* including 10.4% reads related to *“Ca*. Parcubacteria” and 9.1% – to “*Ca*. Saccharibacteria,” which were highly similar to those identified in bottom sediments of Sherlovaya Gora pit lake (Figure 3). *Acidobacteriaceae* were represented in B3 sample by 13.0% of total reads and included several uncultured phylotypes, similar to those identified in Sherlovaya Gora samples. *Mucilaginibacter* spp. (*Sphingobacteriaceae*) were the fourth most-significant group in B3 site (11.9%). Chemoorganotrophic facultative anaerobes of the latter genus were earlier isolated from a range of environments including soils, wetlands, sediments, acidic peat bogs and others (Lee et al., 2015). *Actinobacteria* and *Bacteroidetes* may be responsible for organic matter decomposition and, partially, for iron cycling in Bugdaya ecosystem. Among “*Ca*. Patescibacteria,” formerly known as candidate phylum “*Ca*. Parcubacteria” (OD1) was previously identified as a minor fraction of reads in various anoxic environments such as sediments and rumen, but in relatively high numbers in freshwater ecosystem of Svalbard (Ntougias et al., 2016). This group is considered to include numerous organisms with diverse metabolisms and phylogenies (León-Zayas et al., 2017). “*Ca*. Saccharibacteria” was described to be a cosmopolitan and diverse group with sample-specific phylotypes (Ferrari et al., 2014), confirmed to be present in minor quantities in acidic environments including pit lakes (Falagán et al., 2014; Mendez-Garcia et al., 2015). In the recent SILVA database v.132, this taxon, previously referred to as candidate division TM7, was placed as a family-level subclade within “*Saccharimonadia*” class of candidate phylum *“Ca*. Patescibacteria.” The first cultivated organism related to “*Ca*. Saccharibacteria” group (TM7x) was isolated from the human oral cavity and revealed to possess epibiotic parasitic lifestyle using an actinomycete as a host (He et al., 2015). Considering possible epibiosis between “*Ca*. Saccharibacteria” and actinomycetes, abundance of TM7-related clones in Bugdaya AMD could be attributed to the prevalence of *Actinobacteria* (class *Acidimicrobiia*) in this same ecosystem. However, it is currently hard to consider exact role of “*Ca*. Patescibacteria*”* and, in particular, “*Saccharibacteria*”-related organisms in mine-impacted environments.

Remarkably, the candidate divisions found in Bugdaya were previously detected in Svalbard acid mine drainage-affected area (García-Moyano et al., 2015), which is characterized by comparable parameters: moderate pH and transitionally low temperatures below the freezing point for a significant part of the year. However, while low temperatures are constant in high Arctic, Transbaikalia is situated in the continental climatic zone with outstanding temperature contrasts of about 60°C between summer and winter seasons. Chloroplasts of *Oxyphotobacteria* class (4.5%) from various phototrophic eukaryotes have also been detected in Bugdaya site, being probably washed out from foulings on ore deposits surrounding the sampling spot. Taxa harboring organisms with geochemical activity (generally, sulfur oxidizers, iron oxidizers, and reducers) are only represented as minor components of Bugdaya sample. These organisms were of different phylogenetic affiliations. With the prevalence of iron-oxidizing *Leptospirillum* spp. (1%), their total share was of 2.5% of reads derived from the genus *Ferrithrix* (actinobacteria), genera *Rhodanobacter, Acidiphilium, Acidithiobacillus, Metallibacterium, Gallionella, Sulfuricella* (all, proteobacteria) and genus *Acidicapsa* (from subgroup 1 *Acidobacteriaceae*).

### Natural Acidic Spring Ulan-Bulak

**Figure 4:**
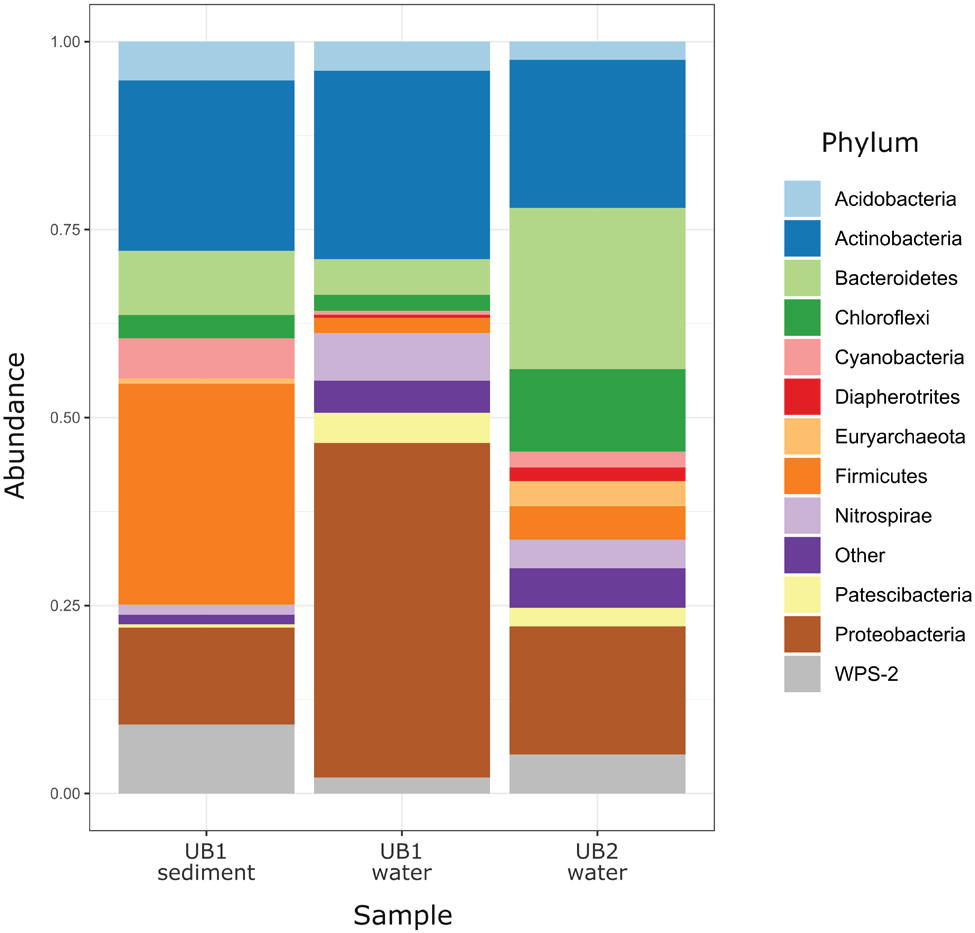
Relative abundance of prokaryotic phyla and candidate divisions in microbial communities of natural acidic spring Ulan Bulak. Low abundance taxa (<1%) are shown together as “Other.”

Community analysis of microbial composition in Ulan-Bulak was performed in two sampling sites (UB1 and UB2), which differed in the thickness of bottom sediment layers (ca. 20 cm thicker in UB1 site, sampled separately) and plant residues amounts correlating with estimated fulvic acids contents (higher in UB2 site as judged by more intense yellow-reddish color of water in this spot). Ulan-Bulak natural acidic spring is characterized as the most acidic environment among all sites studied in this survey (Supplementary Table S3). As a result, a significant proportion of typical acidophilic bacteria known to be involved in iron redox cycling, *Acidithobacillus* spp., *Leptospirillum* spp. and *Acidimicrobiales* have been identified alongside the iron- and sulfur compounds-metabolizing firmicutes (Figures 3, 4). The analysis revealed a high representation in both emplacements of actinobacterial *Acidimicrobiia*-related sequences (without a clear affiliation to cultured representatives) – 15.0, 14.9, and 10.2% for surface water and bottom sediments of UB1 site and water of UB2 site, respectively. In all Ulan-Bulak samples, the taxa comprising iron-oxidizing and -reducing organisms constituted the majority of microbial community in comparison to other, mining-impacted, sites in this study. *Acidithiobacillus*-related sequences were the most significant group in UB1 water sample (23.2%), while in the UB1 sediments, the share of these organisms was smaller (5.0%). Reads related to iron oxidizers of *Leptospirillum* genus (*Nitrospirae*), *Acidithrix* and *Ferrithrix* actinobacterial genera, as well as betaproteobacteria of the genus *Ferritrophicum* totally comprised 13.1% of UB1 water community, while the reads affiliated to the genera *Metallibacterium* (*Gammaproteobacteria*) and *Acidicapsa* (*Acidobacteriaceae* subgroup 1), both of which harbor Fe(III) reducing species, rated at 2.8% in water samples. In the sediments of UB1 site (UB1sed sample), iron oxidizers of the abovementioned groups comprised 5.8% of the community (twice as low as in the water). At the same time, the share of moderately acidophilic *Desulfosporosinus* genus in the sediment was much higher than in UB1 water (up to 6.5% vs. 0.5%). *Desulfosporosinus* (the family *Peptococcaceae*) representatives are known to populate acidic settings, those include few acidophilic sulfate-reducers isolated so far in pure cultures: *D. acidiphilus* from a mining site at Chessy-Les-Mines, France (Alazard et al., 2010), *D. acididurans* from White River sediment in Montserrat (Sánchez-Andrea et al., 2015) and *Desulfosporosinus* sp. strain BG from oxidized mining wastes in Northern Transbaikalia (Karnachuk et al., 2015, 2016). The latter was reported to tolerate high concentrations of copper and produce crystalline copper sulfides (Karnachuk et al., 2015). In another study, the presence of polyvalent metals in ionic form was proposed to mitigate the toxicity of free sulfide in acidic environments by removing it from the aqueous phase through the formation of insoluble sulfides (Meier et al., 2012). Similarly, higher metal ions content together with lower oxygen availability may facilitate proliferation of acidophilic sulfate reducers in UB1 sediments, comparing to the surface water of the site.

Another discrepancy between water and sediment samples of UB1 site is in the composition of putative organotrophic component of the community. In both samples, these organisms were represented by *Paludibacteraceae*-related (*Bacteroidetes*) sequences (4.1% from water and 6.1% from sediment) including those belonging to *Microbacter* and *Paludibacter* genera. However, in water sample UB1, alphaproteobacteria of *Rhizobiales, Sphingomonadales*, and *Caulobacterales* orders, which all harbor aerobic chemoorganotrophic organisms, represent 6.2% of the community, while the sediment sample UB1sed contained much higher percentage of organotrophic *Firmicutes*. Besides the abovementioned *Desulfosporosinus*-related clones, the reads related to families *Clostridiaceae* (group 1), *Lachnospiraceae* and *Ruminococcaceae* comprised 17.5% in the sediment sample. Current state of knowledge on metabolic traits of these *Firmicutes* (Rosenberg et al., 2014) allows predicting fermentative or aerobic organotrophic lifestyle of their representatives in UB1 site. However, the majority of UB1sed *Firmicutes*-related reads is affiliated with uncultured taxa, which role in the ecosystem is unpredictable to the moment.

UB2 sample displayed maximum abundance (20.4%) of sequences related to *Microbacter* genus. The only cultured representative of this genus, anaerobic fermentative propionigenic bacterium *Microbacter margulisiae*, was isolated from the sediment of an acid rock drainage environment at Rio Tinto (Sánchez-Andrea et al., 2014). Notably lower numbers of *Microbacter*-related sequences have been retrieved from UB1 site. Considering carbohydrate- and polypeptide-fermenting lifestyle of these bacteria, the difference in their abundancy in UB1 and UB2 samples correlates with higher organics content of the latter site. Another group represented in the UB2 sample in relatively high proportion (10.7%) was affiliated with uncultured *Chloroflexi* (AD3), previously detected in acidic forest soil, metal-contaminated sites and in an industrial soft coal slags (Selenska-Pobell, 2002; Wegner and Lieasack, 2017), however their metabolic roles remain elusive. Among others, candidate phylum WPS-2 should be named. It contributed 9.2% reads in the sediment sample UB1sed, 2.1% in UB1 water and 5.2% in UB2. Further relatively significant groups in UB2 sample are merged from iron oxidizers of genera *Ferrimicrobium* (3.4%) and *Leptospirillum* (3.8%), as well as from heterotrophs of *Acidibacillus* and *Sphingomonas* genera (2.8 and 2.2%, respectively). The presence of archaeal phylotypes attributed to uncultured TMEG (Terrestrial Miscellaneous Euryarchaeal group, which is referred to as a group of ambiguous taxa in the class *Thermoplasmata* in the recent SILVA database v.132) in numbers 3.0% for UB2 and 0.4% for UB1 sediment (i.e., in both sampling spots of Ulan Bulak) seems rather interesting. These archaea have been found in various environments that also include acidic areas (Mendez-García et al., 2014; Söllinger et al., 2016; Korzhenkov et al., 2019). In UB2 site, OTUs, corresponding to uncultured euryarchaea, clustering together within a separate deep phylogenetic lineage of “DPANN” superphylum, are also considerably represented (1.8%). This lineage has uncertain taxonomic position within the superphylum, being related whether to “*Ca*. Micrarchaea” class-level clade of candidate phylum “*Ca*. Diapherotrites” (Youssef et al., 2015), or to candidate phylum “*Ca*. Micrarchaeota” with equal probability. “DPANN” superphylum includes *Nanoarchaeota* and other archaea with reduced genome sizes and was suggested to have nano-sized cells implying symbiotic or parasitic lifestyle. Recently, interaction of a “*Ca*. Micrarchaeota*”* Mia14 with its archaeal host of *Thermoplasmatales* order was confirmed by using CARD-FISH in the co-culture (Golyshina et al., 2017).

**Figure 5:**
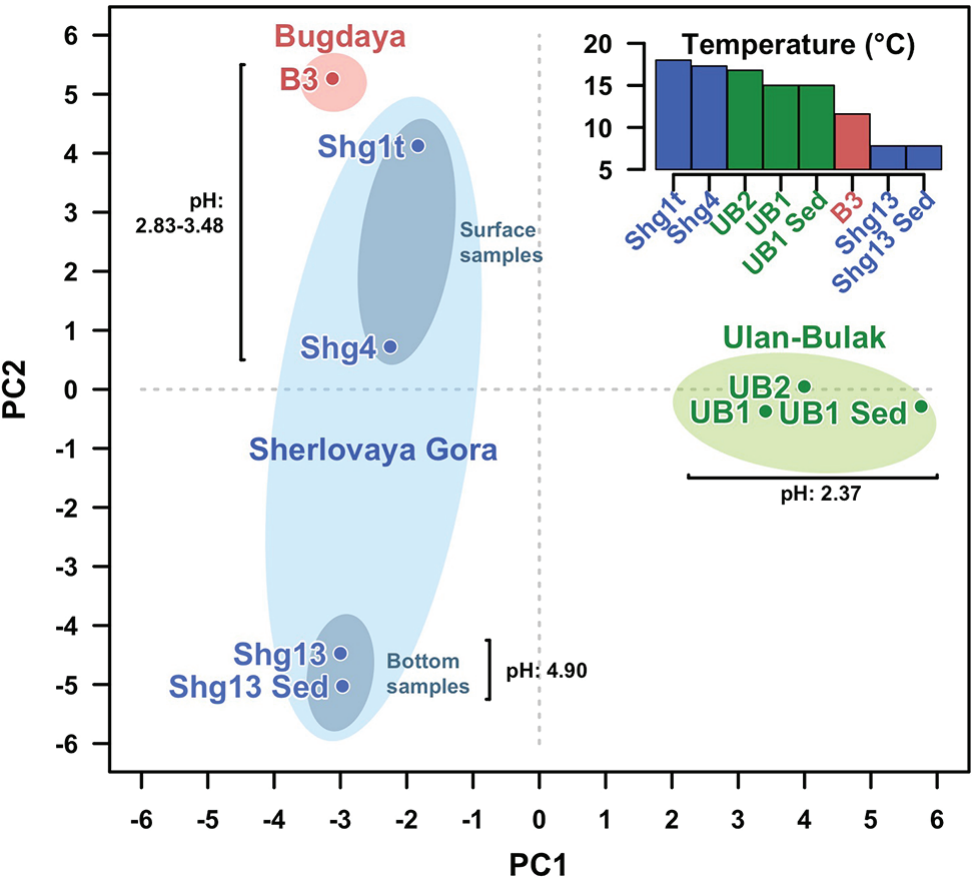
Principal Components Analysis (PCA) clustering all samples from the three sites studied. Fisrt (PC1) and second (PC2) components explain the 26.07 and 24.46% of the variability. Samples from the same site are surrounded by an ellipse of red (Bugdaya), light green (Ulan-Bulak) or light blue (Sherlovaya Gora) colors. Gray ellipses differentiate surface from bottom samples in Sherlovaya Gora. Distances among samples seem to be explained by proximity of the pH. PCA has been developed under *R programming environment* using the function *prcomp* from *stats* package from basic R (R Development Core Team, 2008).

Altogether, the analysis of Ulan Bulak samples indicates that the main components of microbial population in this natural acidic environment are organisms involved in redox transformations of iron, which soluble forms are highly available in the system, and organotrophic organisms degrading complex organic matter entrapped into iron-rich sediments. Comparison of community composition in two sites of Ulan-Bulak with different content of plant residues revealed influence of these organic sources on the distribution of typical acidophiles and novel uncultured groups in this natural acidic environment. A potential for anaerobic acidophilic *Firmicutes* and *Bacteroidetes* as well as for uncultured *Acidimicrobiia* to play a significant role in the community can also be recognized. Principal components analysis (Figure 5) revealed clustering of samples by pH values and temperatures of the sites. Diversity of surface water samples from Sherlovaya Gora pit lake appeared to be more close to that of surface water bodies of Ulan-Bulak and Bugdaya sites than to the bottom samples of the lake, while pH parameter separates all of the samples into three groups, including surface water samples from Sherlovaya Gora and Bugdaya sites, water and sedimentary samples from Ulan-Bulak and bottom water and sediment samples from Sherlovaya Gora pit lake with significantly higher pH values of ca. 5 in contrast to 2.4–3.5 in other sites. Rarefaction analysis across all sampled sites (Supplementary Figure S1) showed that B3 and UB1 are likely to host the most diverse communities spots unlike the Shg4 (microbial mat grown on plant remnants) exhibiting the lowest microbial diversity.

## Conclusion

**Figure 6:**
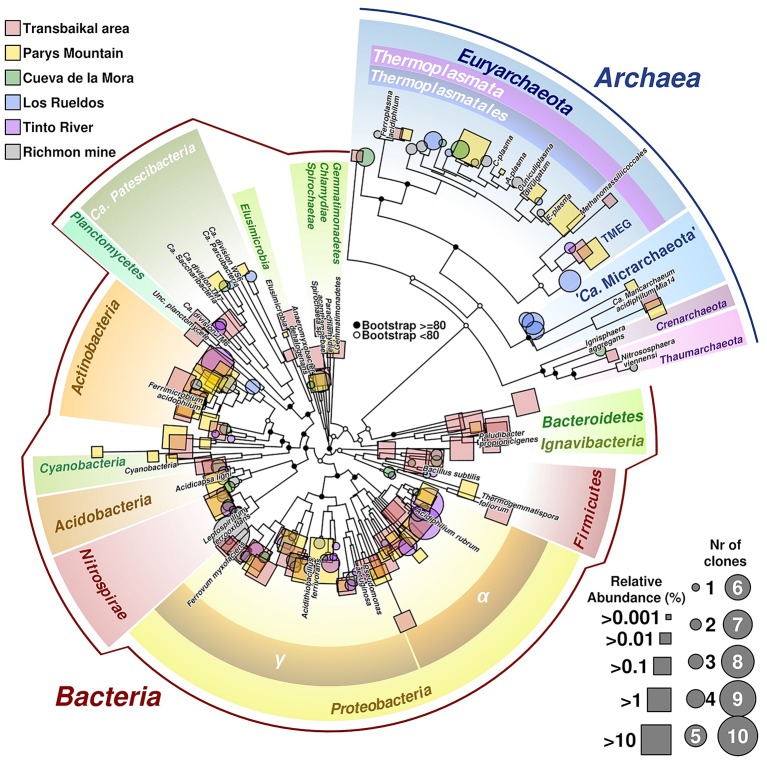
Phylogenetic tree of prokaryotic diversity found in different acidic sites. Sequences belonging to the 16S rRNA gene used in the tree have been obtained from isolated clones (represented in circles) or as reads from barcodes sequencing (squares). Size of circles is related to number of clones of the same taxonomic group found in the site, whereas squares size are related to relative abundance of reads. Labeled branches correspond to reference sequences chosen from each phylum. The tree combines data from different acidic mine drainages (AMDs; Transbailkal area, Parys Mountain and Richmond mine and pit lakes of Transbaikal area, Tinto River and Cueva de la Mora). Sequences have been aligned using *Mafft* (Katoh and Standley, 2013). Resulting multiple alignment was trimmed using *Trimal* (Capella-Gutierrez et al., 2009), removing those columns with more than 50% of gaps or similarity score lower than 0.0001. Phylogenetic tree was calculated by maximum likelihood with bootstrapping of 1,000 replicates. Graphical development was performed under *R* programming environment using basic tools and the package *ape* (Paradis and Schliep, 2018).

Our study has revealed that the composition of microbial communities from these acidic environments with highly contrasting seasonal temperature changes resembles that in other previously studied AMD sites (Figures 3, 6 for a summary on microbial biodiversity). However, in these emplacements some untypical for AMD taxa, e.g. *Ignavibacteriae*, were also detected. Top layers were predominantly populated by photoautotrophic eukaryotes, chemoautotrophic and photoheterotrophic prokaryotes, while the bottom layers were much more diverse with a high proportion of uncultured microorganisms with yet unknown (presumably heterotrophic) type of metabolism. Many of sequences of uncultured taxa originated from candidate phyla and other highly ranked taxa with no previously reported acidophilic members. The question, whether these microorganisms are capable of thriving in a wide range of environmental conditions, or whether this study is a snapshot of a seasonal microbial succession, is widely open and can only be resolved through a continuous monitoring. Nevertheless, the long-term deep freezing of water surface in all studied environments during autumn, winter and spring months, high temperature contrasts between seasons and intensive UV exposure due to very low rainfall and snowfall levels in the area, are the factors shaping microbial communities of investigated extreme environments and making these emplacements unique in comparison to AMD sites studied thus far in temperate climatic zones. Based on their taxonomic affiliation, microorganisms populating these low-pH environments do likely have heterotrophic and iron redox cycling lifestyles. These environments are furthermore of a great potential for exploration of, and prospecting for, biotechnological applications of cold-adapted acidophilic organisms as a source for hydrolases and oxidoreductases, active at low pH and for expanding the toolbox for environmental impact mitigation of AMD.

## Data Availability

The datasets generated for this study can be found in MG-RAST server as a project #mgp88315 and are available by the following link: https://www.mg-rast.org/linkin.cgi?project=mgp88315. MK521704 – MK521803.

## Author Contributions

ST, SG, PG, and OG designed the study. AK and SG did sampling, AK and ST did DNA isolation and sequencing experiments. LZ organized sampling and did chemical analysis. All authors participated in the analysis of the data. OG, SG, and AK wrote the initial draft of the manuscript and revised it with contribution from each co-author.

### Conflict of Interest Statement

The authors declare that the research was conducted in the absence of any commercial or financial relationships that could be construed as a potential conflict of interest.

The reviewer CM declared a past co-authorship with one of the authors OG to the handling editor.
