# Supplementary material for: Microbial Communities of Polymetallic Deposits’ Acidic Ecosystems of Continental Climatic Zone With High Temperature Contrasts
Source: Front Microbiol. 2019 Jul 17;10:1573. doi: 10.3389/fmicb.2019.01573 (PMC6650587; doi:10.3389/fmicb.2019.01573)
Supplement: Supplementary file 1 [file Table_1.DOC]

**Supplementary Material**

**Microbial communities of polymetallic deposits’ acidic ecosystems** **of continental climatic zone with high temperature contrasts**

Sergey N. Gavrilov^*^, Aleksei A. Korzhenkov^*^, Ilya V. Kublanov, Rafael Bargiela, Leonid V. Zamana, Alexandra A. Popova, Stepan V. Toshchakov, Peter N. Golyshin and Olga V. Golyshina ^#^

*these authors contributed equally to the work

**#** corresponding author: [o.golyshina@bangor.ac.uk](mailto:o.golyshina@bangor.ac.uk)

**Supplementary Figures**


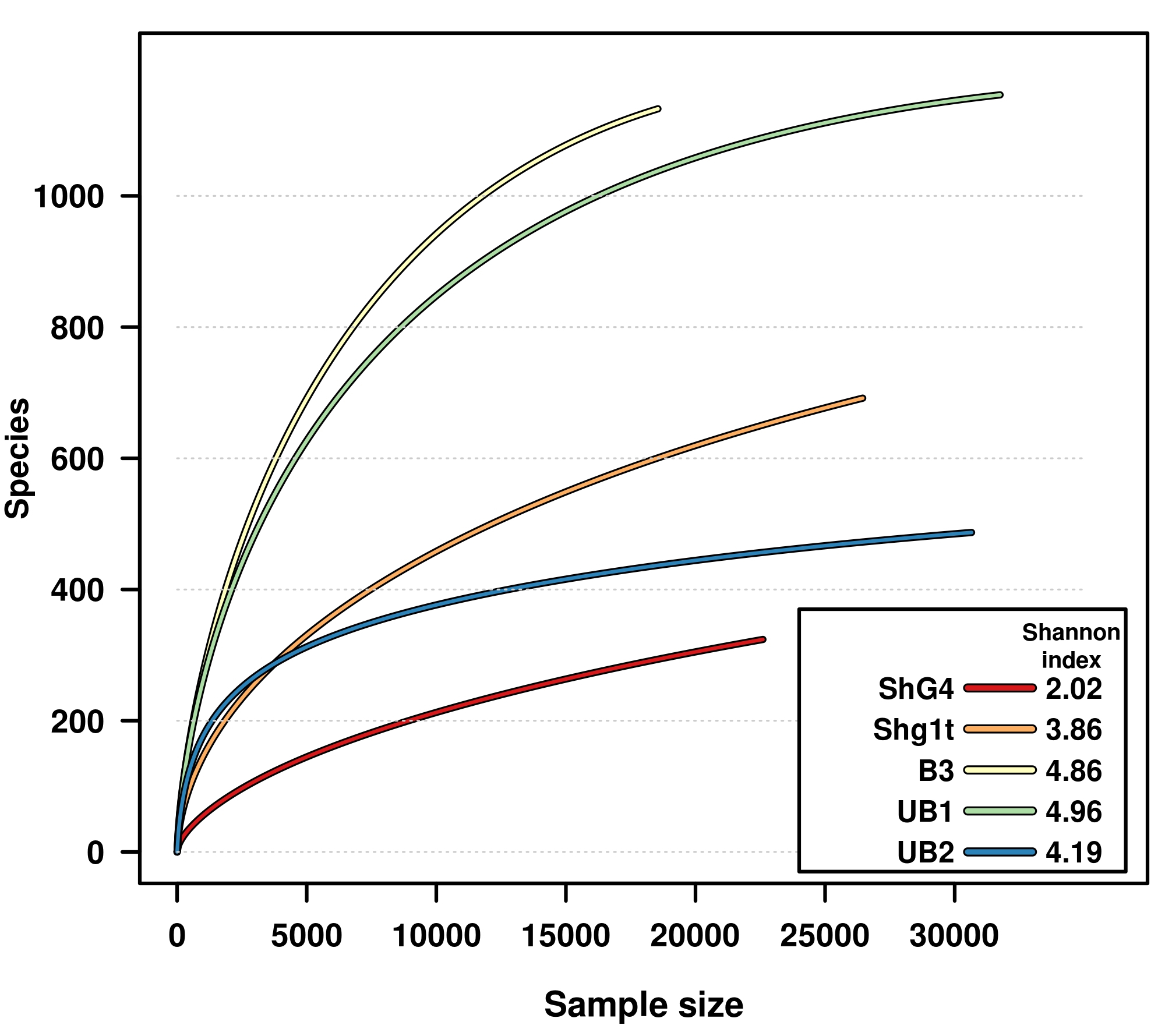


**Figure S1. Rarefaction curves for all the samples of the three different sites.** Shannon index is also shown next to the legend. B3 and UB1 seem to be the most diverse samples, the highest number of species achieved, while Shg4 seems the less diverse. Figure has been developed under *R programming environment [1]* using the package *vegan [2]*.

[1] R Development Core Team (2008). R: A language and environment for statistical computing. R Foundation for Statistical Computing,Vienna, Austria. ISBN 3-900051-07-0;

[2] Dixon, P. (2003). VEGAN, a package of R functions for community ecology. Journal of Vegetation Science. 14(6), 927-930.

**Supplementary Tables**

Table S1. Physico-chemical characteristics of water samples from Sherlovaya Gora pit lake.

| Index | Sample ID | | | Index | Sample ID | | |
| --- | --- | --- | --- | --- | --- | --- | --- |
|  | Shg4 | Shg16* | Shg13 |  | Shg4 | Shg16* | Shg13 |
| Depth, m | Surface | 15.0 | 29.0 | K^+^ | 5.03 | 8.12 | 9.72 |
| T, °C | 17.3 | 8.3 | 9.0 | NH_4_^+^ | 0.48 | 3.59 | 1.22 |
| pH | 2.90 | 3.56 | 4.24 | P_total_ | 0.038 | 0.035 | 0.035 |
| *E_h_*, mV | 567 | 353 | 266 | Si | 26.6 | 38.0 | 39.0 |
| Electrical conductance, mS/cm | 4.09 | 5.04 | 5.04 | Al | 34.47 | 36.28 | 29.74 |
| O_2_ | 0.44 | 0 | 0 | Mn | 95.47 | 139.9 | 138.3 |
| SO_4_^2-^ | 3048 | 4616 | 4750 | Fe | 34.85 | 171.5 | 287.0 |
| Cl^-^ | 5.80 | 4.40 | 4.50 | Co | 1.01 | 1.47 | 1.48 |
| F^-^ | 15.1 | 21.3 | 23.4 | Ni | 3.06 | 3.92 | 3.90 |
| NO_3_^-^ | 0.88 | 1.33 | 0.88 | Cu | 3.02 | 1.27 | 0.357 |
| NO_2_^-^ | 0.02 | 0.042 | 0.03 | Zn | 485.3 | 696.0 | 682.8 |
| Ca^2+^ | 634.0 | 693.4 | 673.6 | Sr | 2.12 | 2.29 | 2.24 |
| Mg^2+^ | 201.9 | 274.5 | 256.4 | Cd | 3.05 | 2.59 | 1.91 |
| Na^+^ | 35.6 | 39.1 | 36.8 | Pb | 0.384 | 0.091 | 0.068 |

Note: Trace elements from Al-Pb – according to ICP MS analysis. * The sample Shg16 shows middle characteristics of the water column, however, this sample was not used for analysis of microbial communities.

**Table S2. Physico-chemical characteristics of water samples from the site B3. Chemical components are represented in mg/l.**

| **Index** | **Value** | **Index** | **Value** | **Index** | **Value** |
| --- | --- | --- | --- | --- | --- |
| T, °C | 11.6 | F^-^ | 77.4 | P_total_ | 0.05 |
| pH | 3.48 | NO_3_^-^ | 2.21 | Si | 35.0 |
| Eh, mV | 511 | NO_2_^-^ | 0.01 | Al | 113.1 |
| Electrical conductance, mS/cm | 2.14 | Ca^2+^ | 206.2 | Mn | 166.2 |
| O_2_ | 8.91 | Mg^2+^ | 48.5 | Fe | 3.02 |
| CO_2_ | 28.6 | Na^+^ | 6.75 | Cu | 10.0 |
| SO_4_^2-^ | 1440 | K^+^ | 3.19 | Zn | 75.4 |
| Cl^-^ | 1.1 | NH_4_^+^ | 1.7 | Cd | 0.55 |

**Table S3. Physico-chemical characteristics of water samples from the site UB1. Chemical components are represented in mg/l.**

| Index | Value |  | Index | Value | Index | Value |
| --- | --- | --- | --- | --- | --- | --- |
| T, °C | 15.0 |  | NO_2_^-^ | 0.02 | Mn | 75.52 |
| pH | 2.37 |  | Ca^2+^ | 697.5 | Fe | 230.0 |
| Eh, mV | 485 |  | Mg^2+^ | 187.8 | Co | 0.707 |
| Electrical conductance, mS/cm | 4.92 |  | Na^+^ | 112.4 | Ni | 1.38 |
| O_2_ | 5.98 |  | K^+^ | 9.96 | Cu | 0.132 |
| SO_4_^2-^ | 3234 |  | NH_4_^+^ | 1.45 | Zn | 4.71 |
| Cl^-^ | 4.9 |  | P_total_ | 0.045 | Sr | 3.50 |
| F^-^ | 1.46 |  | Si | 24.8 | Cd | 0.071 |
| NO_3_^-^ | 2.22 |  | Al | 20.57 | Pb | 0.015 |

Note: Al-Pb – according to ICP MS analysis.
